# Associations between adverse childhood experiences and breastfeeding initiation and duration: a longitudinal birth cohort study in Pakistan

**DOI:** 10.21203/rs.3.rs-7713907/v1

**Published:** 2025-10-21

**Authors:** Emilie E. Egger, Paola Bojorquez-Ramirez, Sarah C. Haight, John A. Gallis, Allison Frost, Siham Sikander, Joanna Maselko, Ashley K. Hagaman

**Affiliations:** University of Pennsylvania Perelman School of Medicine; Harvard T.H. Chan School of Public Health; UNC Chapel Hill Gillings School of Public Health; Duke Global Health Institute; University of North Carolina; University of Liverpool United Kingdom of Great Britain and Northern; University of North Carolina at Chapel Hill; Yale School of Public Health

## Abstract

**Rationale:**

Prior studies have found that a history of adverse childhood experiences (ACEs) is associated with breastfeeding initiation and duration, but the evidence is inconsistent. Despite low- and middle-income countries (LMICs) carrying a disproportionate amount of poor maternal and infant health outcomes, and despite evidence that breastfeeding could alleviate some of these outcomes, little research has assessed the relative impact of ACEs on breastfeeding initiation and duration of exclusive breastfeeding in LMICs.

**Objective:**

The study investigates whether maternal ACEs are associated with breastfeeding initiation and exclusive breastfeeding at 3 and 6 months postpartum among a cohort of women from rural Pakistan.

**Methods:**

The cross-sectional analysis included 869 women 18–45 years of age from the Bachpan Cohort Study. Multivariable modified log-Poisson regression models were used to assess the relationship between any ACE exposure with breastfeeding initiation (n=755) and exclusive breastfeeding at 3 (n=758) and 6 months (n=809). Models were adjusted for known confounders.

**Results:**

55.2% of participants reported experiencing at least 1 ACE. 17.6% of women with a history of at least one ACE reported initiating breastfeeding within an hour of childbirth. 43.8% of women with at least one ACE reported exclusively breastfeeding at 3 months postpartum and 7.6% of women with at least one ACE reported exclusively breastfeeding within the last 24 hours at 6 months postpartum. There was no significant difference between groups for initiating breastfeeding. However, women who had experienced ACEs were less likely to recall exclusively breastfeeding at 3 (RR=0.8; 95 CI=0.7, 0.9) and 6 months (RR=0.8; 95% CI=0.5, 1.2) postpartum compared to women with no self-reported history of ACEs.

**Conclusion:**

Our findings indicate that ACEs may contribute to differences in exclusive breastfeeding. Understanding the long-term effects of ACEs on breastfeeding is important to help inform the design of intervention programs to prevent or mediate the negative repercussions of ACEs.

## INTRODUCTION

Breastfeeding is recommended by the World Health Organization (WHO) as the ideal form of nutrition for infants.^1,2^ In low- and middle-income countries (LMIC) such as Pakistan, breastfeeding can be particularly protective against malnutrition.^3 4 5^More broadly, breast milk has been associated with lower rates of a variety of childhood illnesses like asthma and wheezing.^6 7^ Breastfeeding is also associated with mental and emotional health benefits, such as maternal-child attachment and reduced physiological stress, anxiety, and postpartum depression for breastfeeding mothers.^5^ WHO recommends initiating breastfeeding within one hour of birth.^8^ Initiating breastfeeding within one hour of childbirth is pivotal because it guarantees that infants receive the colostrum— the first breast milk produced for 2–4 days after childbirth.^9^ Compared to other stages of breast milk, colostrum is concentrated with antibodies that provide passive immunity for babies.^9,10^ Initiating breastfeeding within one hour of childbirth has also been shown to be associated with the continuation of exclusive breastfeeding practices.^11^

Breastfeeding initiation and duration of exclusive breastfeeding vary widely across LMIC.^12^ In 2024, nearly two out of three infants were not exclusively breastfed for the recommended six months.^1^ 52% and 54% of women in Pakistan received antenatal counseling about the initiation of breastfeeding and exclusive breastfeeding, respectively.^13^ Counseling rates were highest in urban areas and among women who had relatively high education status.^13^ One in five babies born in Pakistan is breastfed within one hour of birth and 48% are exclusively breastfed at six months old (based on 24-hour recall).^14^ Infant mortality has been declining in Pakistan over the past decade, reaching 62 deaths per 1000 live births in 2018.^13^ Child malnutrition is the cause of nearly 50 percent of these deaths.^15^ Exclusive breastfeeding can reduce infant deaths up to 10 percent.^16^

While many birthing people are aware that breastfeeding provides optimal nutrition benefits for infants, qualitative studies have consistently demonstrated that most are unable to identify the specific benefits associated with breastfeeding.^17^ Many therefore use cultural practices known to reduce hunger and respiratory illnesses in their infants.^15^ For instance, colostrum is sometimes discarded in Pakistan villages because it is perceived as not having value for a child’s wellbeing.^18,19^ In one study, 14% of mothers in urban and semi-urban Pakistan discarded colostrum.^20^ Many Pakistani mothers complementary feed their infants during the first three days of life with water, ghee, animal milk, and herbal tea to optimize newborn nourishment and health outcomes.^18,19,21^ Among children who were breastfed, 76% received pre-lacteal feeds within the first three hours of life.^13^ Pakistani mothers also face the wide variety of common barriers to breastfeeding such as: time management, physiological changes after delivery, perception of insufficient breast milk, high maternal work load, additional maternal stress from initiating breastfeeding early, cesarean section deliveries, employment, and limited workplace support.^18,22^

Adverse childhood experiences (ACEs), categorized as abuse, neglect, and household challenges, are important determinants of maternal and infant well-being .^23,24^ Several studies suggest that the prevalence of ACEs is consistently highest among LMIC countries.^25–28^ ACEs have been found to have an enduring effect on maternal health prior to and during pregnancy.^25^ Evidence suggests that ACEs induce physiological changes that lead to increased risk of poor health and behavioral outcomes.^29,30^ An increase in the number and severity of ACEs also appear to be linked to worse health outcomes throughout the life course, indicating the existence of a dose-response relationship.^31–33^ Evidence suggests that conditions associated with ACEs, such as substance use and interpersonal violence, can reduce the initiation and continuation of breastfeeding, due to higher rates of chronic health conditions and psychosocial difficulties with pregnancy. ^25,34^

Several studies have examined the association between ACEs and breastfeeding outcomes; however, results of these studies vary and few have been conducted in LMIC. One study reported that ACEs are associated with an increased likelihood of exclusive breastfeeding, but not breastfeeding initiation, while another found an association between experiencing child sexual abuse and a higher breastfeeding initiation rate.^23,35^ Another reported a significant association between ACE count and an increased likelihood of initiating breastfeeding, but not continued breastfeeding at two months postpartum.^36^ To our knowledge, no research has assessed the relationship between ACEs and breastfeeding outcomes in Pakistan. This study investigates whether experiencing ACEs is associated with breastfeeding initiation and exclusive breastfeeding at 3 and 6 months among a cohort of women from a rural sub district of Pakistan. Our study tests the associations between ACEs and breastfeeding outcomes in LMIC. Given evidence that breastfeeding can contribute to maternal and infant health, particularly in resource-strained contexts, investigating early life exposures that may hinder breastfeeding uptake may have significant public health impact.

## METHODS

### Study design and participants

Data are from the Bachpan Cohort Study, which enrolled women from rural communities in Kallar Syedan, a subdistrict of Rawalpindi, Pakistan.^37^ The Bachpan Cohort Study is an ongoing prospective pregnancy-birth cohort study designed to investigate the effects of depression on child health and development.^37^ Eligibility criteria for the Bachpan Cohort Study included being married, in the third trimester of pregnancy (≥ 28 weeks’ gestation), at least 18 years of age, intending to remain in the study area, not requiring immediate inpatient care for medical or psychiatric reasons, and being able to speak Urdu, Punjabi, or Potohari.^37^ Within the Bachpan birth cohort is the Thinking Health PLUS Program (THPP), a cluster-randomized trial evaluating a peer-delivered psychosocial treatment for depression.^37^ All eligible pregnant women residing in the study area at the time of recruitment were screened for depression using the Urdu validated Patient Health Questionnaire (PHQ-9).^38^ Women from each of the 40 village clusters who scored a PHQ-9 score ≥ 10 were defined as screening positive for depression and were invited to join the Bachpan Cohort, while one of every three women who did not meet the threshold were invited to participate as the non-depressed reference group.^37^ The ≥ 10 threshold was used to enroll pregnant women into the trial portion of the cohort. A total of 1154 pregnant women were enrolled into the cohort and complete baseline between October 2014 and February 2016.^37^ Additional eligibility for the present study required available data on ACEs, breastfeeding initiation, and breastfeeding continuation. Data from the following timepoints were utilized in this analysis: third trimester during pregnancy (baseline), 3 months, 6 months, and 36 months postpartum.

### Measures

#### Outcomes

The three primary outcomes of interest were selected based on the World Health Organization breastfeeding recommendations to initiate breastfeeding within the first hour of birth and exclusively breastfeed for the first 6 months of life: (1) time of breastfeeding initiation (“When was the baby’s first breast milk feed?”) and exclusive breastfeeding at 3 and 6 months (“What has the child had in the last 24 hours?).”^8^ Children who were only fed breast milk in the last 24 hours (no reported feeding of ghutti, herbal water, water, or tea) were categorized as having been exclusively breastfed. Each of the three outcome variables were treated as binary variables (yes/no).

#### Exposure

The ACE questionnaire is used to measure experiences of abuse, neglect, household dysfunction, and community dysfunction during childhood.^24^ ACEs were measured using the 12-item ACE-IQ at 36-months postpartum and retrospectively asks about an individual’s exposure to adverse experiences prior to the age of 18. This questionnaire has been validated for use in international settings and was additionally adapted for use in the Bachpan Cohort Study.^24^ Sexual abuse questions were removed because of the high probability of underreporting due to stigma and fear in the community.^24^

ACEs were operationally defined in three ways.^24^ ACEs variables included (1) a binary yes/no variable for any ACE reported (2) a categorical ACE variable for the number of experiences reported (0,1, 2, 3, 4+), and (3) a binary indicator for each of the four ACE domains ( (1) neglect (emotional neglect; physical neglect), (2) family psychological distress (alcohol and/or drug abuser in the household; incarcerated household member; someone depressed, mentally ill, institutionalized or suicidal), (3) home violence (physical abuse; emotional abuse; household member treated violently), and (4) community violence (bullying; community violence; collective violence). Binary variables for each ACE domain were coded ‘yes’ if a woman experienced any of the ACEs within the domain.

#### Control Variables

All statistical models described below were adjusted for mother’s age at baseline, education level (> 5th grade) as a proxy for childhood socioeconomic status,^39^ number of living children,^40^ trial arm (control, intervention, non-depressed, which could be related to childhood experience of mental health), assessor (at baseline and at 36 months because of potential influence on responses to breastfeeding and ACEs), and baseline predictors of missingness by p < 0.10 at the respective waves due to participants leaving the study.^39,41^ While we do not believe these variables to be confounders, we included them to minimize the variance in the outcome that is not explained by the main exposure. In this case, we believe the proposed variables may be strongly related to reporting of ACEs and breastfeeding outcomes and adjusting for these variables can increase efficiency (i.e., more narrow confidence intervals). No baseline variables were associated with missingness at 3 months. Baseline variables associated with missingness at 6 months were an asset-based SES variable created using polychoric PCA, total number of people per room, grandmother living in child’s home (yes/no), and categorical variable of living children (first pregnancy, 1–3 children or 4 + children). The trial arm was associated with missingness at 36 months. Baseline variables associated with missingness at 36 months are a categorical variable for the statement “My faith gives me strength” (no, sometimes, yes) and the total number of people per room.^41^

#### Statistical Analyses

Multivariable modified log-Poisson regression models with cluster robust standard errors and exchangeable working correlations in Stata were used to assess the relationship between each type of ACE variable and the three respective outcomes— breastfeeding initiation within the first 24 hours of childbirth (n = 755; measured at 3 months), 24 hour recall of exclusive breastfeeding at 3 months (n = 758), and 24 hour recall of exclusive breastfeeding at 6 months ( n = 809). 24-hour recall is a WHO-accepted indicator for retrospective questions about breastfeeding exclusivity at 3 and 6 months.^42^ The use of modified log-Poisson models, instead of logistic regression models, were the analysis of choice because the misinterpretation of odds ratios as risk ratios persists in global health research with common outcomes such as ours.^43^ This is especially important in global health research, which often measures binary outcomes for which overstating associations of risk can be especially costly and dangerous.^43^ Moreover, the use of robust standard errors account for the misspecification of the outcome distribution (i.e. Poisson instead of binomial distribution) and allow researchers to report valid risk ratios for binary outcomes.^43^ Statistical analyses were performed in Stata 15.1.

#### Ethics

This study was approved by institutional review boards at the Human Development Research Foundation (Pakistan), the University of North Carolina at Chapel Hill (United States), and Duke University (United States). Written informed consent was required for study participation and was acknowledged with the participant’s signature or by a witness’s signature in the case of the participant being illiterate. Research was conducted in accordance with the “Common Rule” set out in the Belmont Report and the Declaration of Helsinki.

## RESULTS

### Descriptive Statistics

Of the total 1154 women enrolled at baseline, 869 answered questions about ACE data at 36 months, resulting in 755, 758, and 809 women for each of our outcomes—breastfeeding initiation, and exclusive breastfeeding at 3 and 6 months, respectively. Table 1 displays demographic and breastfeeding characteristics of the Bachpan Cohort stratified by history of ACE exposure (yes/no). Nearly 60% of women in the cohort reported experiencing at least one ACE domain.^24^ Women with an ACE history on average had a greater number of living children (1.5 vs. 1.4 children), and greater household size (8.6 vs. 8.3 children and adults) than women with no ACE history. Most women had a primary education greater than 5th grade (66.7%). Overall,15.6% of women in the study initiated breastfeeding within one hour of birth. 47.9% reported exclusively breastfeeding at 3 months and 8.7% reported exclusively breastfeeding at 6 months.

### ACEs and Breastfeeding Initiation

A greater percentage of women with an ACE history reported initiating breastfeeding within an hour of giving birth compared to women with no ACE history (17.5% vs. 13.4%).

Table 2 presents statistics stratified by the number and percentage of women who either did or did not report initiating breastfeeding within one hour of childbirth for each operationalization of ACE. Among women who reported initiating breastfeeding within one hour of childbirth, 61.9% had a history of ACEs, compared to 54.2% of women who did not report initiating breastfeeding within one hour of childbirth. The average number of ACEs among women who initiated breastfeeding within one hour of childbirth is 1.2, while the average number of ACEs among women who did not initiate breastfeeding within one hour of childbirth is 1.1. The most common ACEs among women who did not report initiating breastfeeding within one hour of childbirth were (1) emotional abuse (31.1%), (2) physical abuse (22.8%), and (3) household members being treated violently (12.4%). Women who did initiate breastfeeding within an hour of childbirth also reported emotional abuse and physical abuse as the most common ACEs, but reported one or no parents, parental separation, or divorce (13.6%) as the third highest.

Panel 1 in Table 5 shows the adjusted relative risks (ARR) between each ACE domain exposure and breastfeeding initiation within an hour of childbirth. We found no significant risk associated between any of the ACE type variables and breastfeeding initiation (Models 1–10). Although the confidence intervals are wide and include the null value, there was a suggestive trend that women exposed to ACEs were more likely to have initiated breastfeeding within an hour of giving birth compared to women with no history of ACEs (ARR=1.2; 95% CI: 0.8, 1.8).

### ACEs and 24-Hour Recall of Exclusive Breastfeeding at 3 Months Postpartum

Less than half of women with an ACE history (43.8%) reported exclusively breastfeeding in the previous 24 hours at the 3-month postpartum visit, while over half of women with no ACE history (53.0%) reported exclusively breastfeeding in the previous 24 hours at the 3-month postpartum visit. Table 3 presents statistics stratified by the number and percentage of women who either did or did not report exclusively breastfeeding during the 3-month postpartum visit for each type of ACE variable. Most women who exclusively breastfed and did not exclusively breastfeed their child for the last 24 hours had a history of ACEs (50.4% vs. 59.5%, respectively). The average number of ACEs among women who exclusively breastfed is 0.98, while the average number of ACEs among women who did not exclusively breastfeed their child is 1.18. The most common ACEs among women who did not exclusively breastfeed were (1) emotional abuse (33.16%), (2) physical abuse (24.30%), and (3) emotional neglect (13.67%). This was similar to those who did recall breastfeeding, but those women reported household member/s were treated violently (11.02%) as the third highest exposure.

Panel 2 in Table 5 shows the ARR between each ACE domain exposure and 24-hour recall of exclusive breastfeeding at 3 months postpartum. Table 6b shows RR between ACE and outcome. Women exposed to ACEs were less likely to have exclusively breastfed compared to women with no ACE exposure (ARR=0.8; 95% CI: 0.7, 0.9). Total ACE Score, ACE categorical, and the community violence domain were also associated with 24-hour recall of exclusive breastfeeding at 3 months postpartum (Model 5, 6, and 10, respectively). With every additional exposure to an ACE, women were 10% less likely to have reported exclusively breastfeeding (ARR=0.9; 95% CI: 0.9, 1.0). Women exposed to one ACE were 20% less likely to have exclusively breastfed for the last 24 hours (ARR=0.8; 95% CI: 0.6, 1.0) than women not exposed to ACEs. We found no significant association between any of the other ACE type variables and exclusively breastfeeding at 3 months postpartum (Models 2–4 and 7–9).

### ACEs and 24-Hour Recall of Exclusive Breastfeeding at 6 Months Postpartum

Twenty-four hour recall of exclusive breastfeeding at the 6-month postpartum visit for women with and without an ACE history was lower by nearly 20% (specifically 17.45% and 18.83%, respectively) compared to 24-hour recall percentages of exclusive breastfeeding at 3 months. Table 4 presents statistics stratified by the number and percentage of women who either did or did not report exclusively breastfeeding their child within the last 24 hours during the 6-month postpartum visit for each type of ACE variable. Most women who exclusively breastfed and did not exclusively breastfeed their child for the last 24 hours had a history of ACEs (50.0% vs. 57.2%, respectively). The average number of ACEs among women who exclusively breastfed their child for the last 24 hours is 0.96, while the average number of ACEs among women who did not exclusively breastfeed their child for the last 24 hours is 1.15.

The most common ACE domains among women who did not exclusively breastfeed for the last 24 hours were (1) emotional abuse (32.07%), (2) physical abuse (23.55%), and (3) emotional neglect (14.21%), similar to women who did exclusively breastfeed for the last 24 hours. Similar to the results seen for 24-hour recall of exclusive breastfeeding at 3 months, nearly one third of women who exclusively breastfed and over one third of women who did not exclusively breastfeed for the last 24 hours at 6 months experienced home violence (32.86% vs 37.75%, respectively).

Panel 3 in Table 5 shows the ARR between each ACE type variable exposure and 24 hour recall of exclusive breastfeeding at 6 months postpartum. There were no significant risks associated between any of the ACE type variables and 24-hour recall of exclusive breastfeeding (Models 1–10). Table 6c shows RR between ACE and outcome. Although not statistically significant, women exposed to ACEs were 20% less likely to have exclusively breastfed for the last 24 hours (ARR=0.8; 95% CI: 0.5, 1.2) than women not exposed to ACEs.

## DISCUSSION

Our study provides novel evidence suggesting that the experiences of ACEs in Pakistan could be associated with lower breastfeeding rates at three and six months postpartum. The findings indicate that mothers with a history of ACEs, increased exposure to ACEs, and exposure to community violence are significantly less likely to report exclusively breastfeeding at 3 months postpartum. We did not find an association between the experience of ACEs and breastfeeding initiation. To our knowledge, this is the first study to assess the relationship between ACEs operationalized as different variables with breastfeeding outcomes in rural Pakistan. The most common ACEs experienced among women in this study fall within the home violence domain (specifically, physical and emotional abuse).

Our study found a significant impact of ACEs on exclusively breastfeeding at 3 and 6 months postpartum. Notably, at 3 months postpartum, each additional ACE was associated with a 10% lower likelihood of exclusively breastfeeding in the past 24 hours (ARR = 0.9; 95% CI: 0.9, 1.0). The study also found that the percentage of women reporting exclusive breastfeeding in the past 24 hours at 3 months postpartum—regardless of ACE history—was more than five times higher than at 6 months postpartum which is the WHO’s recommended duration for exclusive breastfeeding.^2^ Our study builds on prior research linking ACEs to shorter breastfeeding duration by suggesting a cumulative effect of ACEs on breastfeeding outcomes. One study in Bangladesh also showed a dose-response relationship between childhood maltreatment and rates of exclusive breastfeeding. Unlike the previous study, our study used “point in time” data collection at two data collection points.^44^ It also used ACE-IQ an adapted standardized, multi-item measure to assess maltreatment. Our findings differ from a previous study that found a higher association between ACE count and breastfeeding initiation, but none in breastfeeding exclusivity at two months postpartum.^36^ However, our study was conducted in an LMIC, which may suggest that the relationship between ACE count and breastfeeding outcomes varies by context.^36^ This finding warrants research on the relationship between ACE count and breastfeeding outcomes in other South Asian countries and LMIC to understand this relationship.

Our study also contributes to the burgeoning scholarship on ACEs in LMIC by identifying which types of ACEs may be more prevalent in these settings and highlighting the usefulness of the ACE framework across diverse contexts.^26^ The ACE-IQ may not capture child adversity in its entirety.^45–48^ There is currently no way for the measure to account for protective factors against childhood adversity, such as stable emotional relationships with family and community members or how children build resilience.^45,49^ Furthermore, various populations may conceptualize adversity and hardship differently.^50^

Our study found that community violence was associated with a lower rate of exclusive breastfeeding at three months postpartum. Our study adds to research that suggests that experiencing forms of violence can decrease breastfeeding initiation, exclusivity, and duration.^51,52^ Interventions to support women experiencing interpersonal violence during the postpartum period have been successful in reducing violence and depression.^53,54^ Some of this research has been conducted in LMIC.^55^ However, the relationship between experiencing violence and breastfeeding can differ depending on racial identity and type of violence experience.^56^ Future research could investigate the mechanisms that lead to decreased breastfeeding initiation, exclusivity, and duration among women who have experienced community violence as children and could address whether providing therapeutic and breastfeeding support could increase these metrics. For example, breastfeeding promotion interventions could include peer support groups for women who have experienced neglect, sexual assault, or who are currently experiencing domestic violence.^57,58^ Breastfeeding interventions can also serve as a bridge to social services.^59^ Additionally, women in Pakistan who have experienced ACEs often experience higher rates of depression and anxiety.^24^

The high prevalence of exposure to physical and emotional abuse at home as children among this cohort may explain why exposure to ACEs is associated with lower likelihoods of exclusive breastfeeding recall at 3 months postpartum. Physical and emotional abuse experienced during adolescence has been found to be associated with risk of adult intimate domestic violence.^60–63^ Continued violence and an unsupportive environment at home may mitigate any of the potential mental health benefits that breastfeeding may have; a 2024 study in Pakistan noted that women who experienced intimate partner violence were less likely to initiate breastfeeding and exclusively breastfeed.^64^ The integration of social services to address the high prevalence of abuse that many children may be experiencing needs to be prioritized to prevent home abuse from occurring.^65^ The compilation and distribution of resources for children and families experiencing abuse are also needed to strengthen communities and mitigate the negative effects of ACEs later in life. Additionally, future research exploring how experiencing childhood sexual abuse may influence breastfeeding outcomes in rural Pakistan is warranted, especially given the high prevalence and underreporting of such abuse.^44,66–68^

Our study suggests that more research should investigate cultural facilitators to breastfeeding, such as antenatal counseling on the importance of breastfeeding or living in an area where initiating breastfeed is a cultural norm.Culturally safe breastfeeding promotion programs should be developed to address the pervasive misunderstandings and myths that exist about breastfeeding in Pakistan so that women can make informed decisions about their breastfeeding practices and receive desired support.^15,18,69^ When birthing people are cared for in a culturally safe way, they tend to experience better perinatal outcomes and to also continue with recommended care.^70–73^ Breastfeeding promotion support groups could be a way to integrate health services that address domestic violence, food insecurity, and generally build positive community support, especially in areas in which prenatal breastfeeding counseling is prominent, such as Pakistan.^74^

### Strengths and Limitations

Our study has several strengths. First, our study is the first to assess the relationship between ACEs and breastfeeding outcomes in Pakistan. The study also used an ACE questionnaire that has been validated for LMIC settings. This study also provides descriptive statistics about the prevalence of breastfeeding initiation and exclusive breastfeeding recall among women in rural Pakistan which to our knowledge is not available elsewhere. Additionally, the study collected breastfeeding data at multiple time points and employed multiple operationalizations of ACEs.

There are limitations in this study that warrant discussion. First, the exposures (ACEs) were assessed after the outcomes. This is typical of ACE reporting because the ACE questionnaire is a retrospective assessment of childhood experiences that irrespective of which time point they had been assessed for in the Bachpan Cohort could not be captured in real time. However, ACEs is the only validated questionnaire that attempts to capture adverse childhood events among adults. Breastfeeding outcomes are self-reported and there is the potential to overreport breastfeeding recall due to social desirability bias. Another limitation is that exclusive breastfeeding was only measured in the last 24 hours. Although education level was adjusted for as a proxy for childhood SES, there is a possibility of residual confounding by childhood SES. Moreover, findings within ACE domains (particularly, the community violence domain) should be interpreted with caution due to small sample sizes.

Furthermore, not all women with ACE data were assessed for each of the respective outcomes included in this study. There were 114, 111, and 80 missing women for breastfeeding initiation and exclusive breastfeeding recall at 3 and 6 months, respectively. There may be potential differences between participants with ACE data included at baseline and women who were missing which would introduce selection bias. Importantly, variables associated with missingness at baseline and 6 months were controlled for in all regression models.

The Bachpan Cohort had a higher reported prevalence of ACEs compared to most high-income countries but lower prevalence of ACEs compared to other LMIC.^24^ It is important to consider that the prevalence of ACEs in this cohort may be underestimated. The potential underestimated prevalence of ACEs in this study may explain the wide confidence intervals between most ACE type variables with each breastfeeding outcome and no clear dose-response relationship. This information is crucial because a study of women of Bangladesh found that the type of maltreatment experienced affected exclusive breastfeeding differently: women who experienced childhood sexual abuse, specifically, had significantly lower likelihoods of breastfeeding their children compared to women who were not sexually abused as children.^75^ According to this information, an underestimation of ACE exposure in this study would result in lower estimated associations toward the null.

## CONCLUSION

ACEs are a serious public health concern that may have long term repercussions on overall well-being across the life course. We found that for each additional ACE exposure, women were 10% less likely to have reported exclusively breastfeeding for the last 24 hours at 3 months postpartum and that experiencing community violence as a child was also associated with lower rates of exclusive breastfeeding at 3 months postpartum. It is important to identify interventions that can prevent the occurrence of ACEs in rural Pakistan that can negatively impact a mother’s well-being and child’s development. Integration of a life course perspective in medical settings can ensure women are connected to and have access to resources they need to be well and provide good care to their children. It is worth considering looking into medical screenings of ACEs to determine whether this is a viable recommendation. Women can be screened, counseled and linked to support. The findings of this study also help understand the prevalence of breastfeeding outcomes in rural Pakistan. Understanding the long-term consequences of ACEs for women among a population that is already overburdened with other challenges is important for the development of tailored interventions that support the health of women and their children.

## Supplementary Material

Supplementary Files

This is a list of supplementary files associated with this preprint. Click to download.

• Tables.docx

## Data Availability

Data will be released upon completion of the study and, additionally, are available from the senior author Maselko on reasonable request.
